# *Diospyros montana* mediated reduction, stabilization, and characterization of silver nanoparticles and evaluation of their mosquitocidal potentiality against dengue vector *Aedes albopictus*

**DOI:** 10.1038/s41598-023-44442-7

**Published:** 2023-10-11

**Authors:** Rajesh Kumar Malla, Goutam Chandra

**Affiliations:** Mosquito Microbiology and Nanotechnology Research Units, Parasitology Laboratory, Department of Zoology, The University of Burdwan, Burdwan, 713104 West Bengal India

**Keywords:** Zoology, Nanoscience and technology

## Abstract

Recent research has focused on nanoparticles. *Aedes albopictus* is a potential vector that transmits fatal diseases. Recently, Phyto-reduced silver nanoparticles (AgNPs) were shown to be mosquito larvicides. This study aimed to synthesize silver nanoparticles using *Diospyros montana* leaf extract, characterize them, and test their efficacy as larvicide and pupicide against *Ae. albopictus* mosquitoes, determine their duration of effectiveness as a larvicide, identify plant compounds that help to synthesize nanoparticles, and assess their effects on non-target organisms. Quercetin, luteolin, kaempferol, gallocatechin gallate, epigallocatechin gallate, and capsaicin are among the novel reducing and capping agents found in *D. montana* leaf through LCMS analysis. The color shift and distinctive peak in UV–Vis spectroscopy made it simple to see how biogenic AgNPs were produced by converting Ag^+^ ions into Ag^0^. Substantial negative value (− 19.10 mv) of zeta potential demonstrated the long-term stability of AgNPs. A moderate range (8.72 − 50.75 nm) of particle size distribution pattern was obtained using the DLS technique. SEM and TEM images depicted the quasi-spherical (or polyhedral) and spherical shape of the nanoparticles, having approximately 16.75 nm average size. Synthesized AgNPs had a low LC_90_ value (< 10 ppm) for all larval instars and pupae of *Ae. albopictus* and had negligible mal effect on non-target organisms. Regression equations showed dose-dependent mortality by the positive correlation between mortality rate and AgNPs concentration, and each time the regression coefficient (R^2^) value was larger than zero. This study shows that *D. montana* leaf extract is an environment-friendly and sustainable source of an effective reducing and capping agent to synthesize highly stable, ecologically acceptable silver nanoparticles and their application as mosquitocide.

## Introduction

The study of nanoparticles is the focus of recent scientific research on chemical biology. Nanoparticles are particles of atomic and molecular scale, ranging from 1 to 100 nm in size. They possess various intriguing physical, biological, catalytic, and optical properties that have piqued the interest of modern advanced research thinking. Different physical, chemical, and biological methods have produced various types of metal nanoparticles. Silver nanoparticles (AgNPs) are widely synthesized due to their extensive applications and area of use perspective. Production of silver nanoparticles using various chemicals, namely hydrazine hydrate, citrate, sodium borohydride, DMF, ethylene glycol, etc., as reducing agents may lead to the absorption of hazardous substances on the surfaces of nanoparticles, generating toxicity issues^1^. Also, these methods are relatively more expensive^2^. Synthesis of nanoparticles employing a green root, i.e., using a plant-based material as both a reducing agent and capping agent, is comparatively less toxic and cost-effective^3^. Different plant primary and secondary metabolites, such as carbohydrates, proteins, terpenoids, and alkaloids, have been reported as reducing agents of AgNO_3_ for the green synthesis of metallic nanoparticles. Terpenoids, Alkaloids, phenolic acid, and polyphenol compounds like flavonoids are the major bioactive phytochemicals that assist in such processes due to their substantial reducing and stabilizing properties^4^. Silver nanoparticles exhibit size and shape-dependent unique properties which are of interest for applications in different fields such as medical imaging^5^, filters, drug delivery, sensor technology^6^, optical devices^7^, biological labeling^8^, catalysis^9^, molecular recognition and diagnosis^10^. Recently, Ag nanoparticles have also been applied to harmful biological organisms as antibacterial^11^, antiviral^12^, antifungal^13^, anti-inflammatory^14^, anticancer^15^ and mosquitocidal^16^ agents.

*Aedes* is a threatening genus of mosquito vectors that spread several death-dealing viral diseases, including dengue, yellow fever, zika virus, chikungunya, etc.^17^, and found in all tropical, subtropical, and temperate regions throughout the world. An estimated 390 million dengue virus infections occur yearly, of which 96 million manifests clinically^18^. According to WHO (2019), 47 countries (34 in Africa and 13 in Central and South America) are either endemic for yellow fever. As of 2023, 34 African countries and 13 Central and South American countries are endemic for yellow fever or have regions that are endemic for yellow fever^19^. WHO^20^ also announced that 86 countries and territories had reported evidence of Zika virus infection transmitted by mosquito vectors. According to the European Centre for Disease Prevention and Control, in 2022, and as of 24 August, 229,029 cases and 41 deaths from chikungunya have been reported^21^. Such alarming increases in vector-borne diseases demand control of the vector population because that is the principal and most effective method to combat the spreading of these diseases^22^. However, challenges for mosquito vector control are the choice and availability of effective, economical, and secured insecticides. Most of the insecticides available in the market are synthetic chemicals, which are of high cost. Their frequent unscientific applications have many unexpected implications, including the production of resistant strains of mosquitoes, the elimination of non-target organisms, and the establishment of ecological imbalance in the environment^23^. Because of these disadvantages, there is a definite requirement for alternative control methods which may replace these synthetic insecticides. Green synthesized nanoparticles using plant extract may be a suitable alternative for vector control strategies^24^. Recently, the use of phyto-reduced silver nanoparticles as mosquito larvicides has been reported^25^.

*Diospyros montana* Roxb. The Bombay ebony is a small deciduous, dicotyledonous tree under the family Ebenaceae. This plant is a naturally occurring deciduous forest tree widely distributed throughout India. It is used in traditional Indian medicines, including Ayurveda and Unani. Parts of this plant are used in treating several diseases, particularly in folk medicine and also for treating anti-inflammatory, anticancer, etc.^26^. Crude extract from the leaves of this plant also showed mosquito larvicidal properties^27^.

The present study was aimed at synthesizing silver nanoparticles using leaf extract of *D. montana* as a reducing as well as stabilizing agent, characterization of the synthesized nanoparticles, evaluation of its efficacy as larvicidal and pupicidal agent against *Aedes albopictus* mosquitoes, Examination of the length of its efficacy as mosquito larvicide, identification of plant compounds those assist the synthesis process of metal nanoparticles and assessment of its influence on different non-target organisms.

## Materials and methods

### Chemicals

The experiments used silver nitrate (AgNO_3_) acquired from Merck, double distilled water, and normal saline.

### Collection and identification of plants

**Figure 1:**
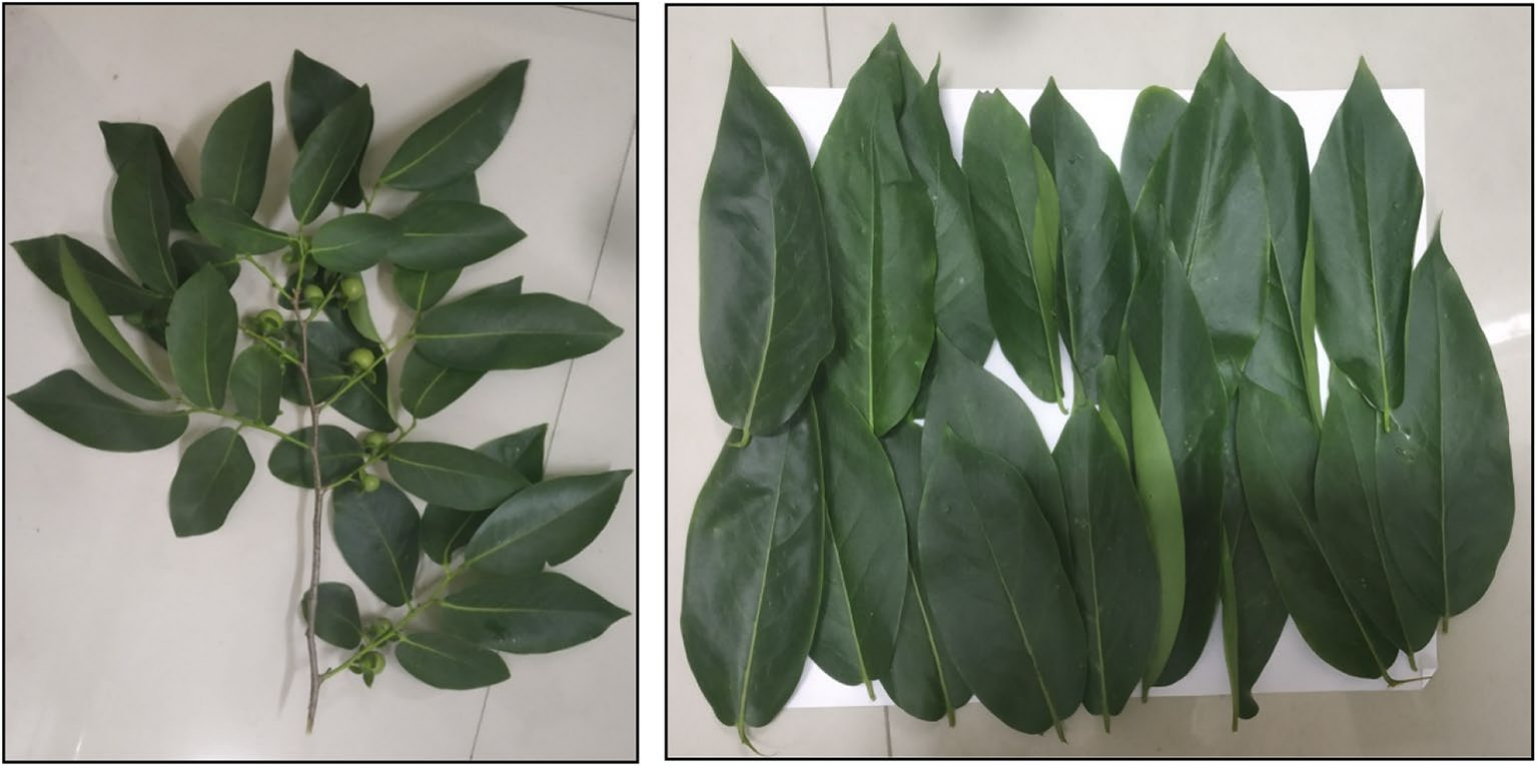
Collected leaves of *Diospyros montana* during the study.

Fresh and mature leaves of *D. montana*, a native plant to India with cosmopolitan distribution, were collected from Ramnabagan Wildlife Sanctuary (23°16′ N, 87°54′), adjacent to the Golapbag campus of the University of Burdwan (Fig. 1). As an endemic species, this plant is available in good numbers on our university campus. The use of *D. montana* does not violate the local regulations of India. During the sample collection from a specific plant, permission was taken from the authority of the sanctuary. Prof. Ambarish Mukherjee, Department of Botany, The University of Burdwan, identified the plant. A herbarium sheet bearing reference No (GCRKM/2017/S011) of the plant was preserved in the Department of Zoology, The University of Burdwan.

#### Complies with international, national and/or institutional guidelines

Experimental research and field studies on plants, including the collection of plant material, comply with relevant institutional, national, and international guidelines and legislation. Studies of the plant (*Diospyros montana*) were carried out in accordance with relevant institutional, national, and international guidelines and legislation.

### Collections of mosquito larvae and culture

Larvae of *Ae. albopictus* were collected from rainwater-dampened tires along the side road and small water-filled waste containers. Larvae were maintained in the culture trays filled one-third with chlorine-free tap water. Artificial food was prepared by mixing dog biscuits and yeast extract in a 1:3 ratio and supplied. The amount of food was 0.5 mg/larva. A temperature of 27 ± 2 °C, a humidity of 85%, and a 14:10 light–dark hour condition were maintained to ensure their metamorphosis^28^. After emergence, adult mosquitoes were allowed to imbibe blood meal from the pigeon. Adult *Aedes albopictus* mosquitoes were identified according to the key of Chandra^29^. Adults were allowed to lay eggs in the water-filled small container in the mosquito cage. Immatures hatched from eggs were used for mosquito larvicidal and pupicidal bioassays.

### Preparation of aqueous phytoextract and its phytochemicals analysis

Leaves were cleaned under the running cold tap water to remove unwanted dust and litter. Washed leaves were allowed to be air-dried for 30 min. Ten grams of fresh leaves were taken and cut into small pieces with scissors. Small amounts of plant leaves were mixed with 200 ml of double distilled water in a glass beaker to prepare aqueous phytoextract. The mixture was warmed in a hot water bath for 10 min at 50 °C and then allowed for rigorous shaking in a magnetic stirrer for an hour. A yellowish-green extract was filtered from the mixture by a Whatman filter paper no 1 and used to synthesize silver nanoparticles. The extract was also subjected to qualitative phytochemical analysis for the identification of primary and secondary metabolites using standard methods of Harborne (1984), Stahl (1989), Trease and Evans (1989), and Sofowara (1993) with slight alterations as follows^30–32^. To detect plant compounds responsible for the reduction of AgNO_3_ and stabilization of nanoparticles, Liquid chromatography-mass spectrometry (LCMS) analysis of the leaf extract was performed.

### Synthesis, separation, and collection of silver nanoparticles

25-mmol AgNO_3_ stock solution was prepared by mixing AgNO_3_ crystals and 100 ml of double distilled water. Twenty-five milliliters of phytoextract of *D. montana* was mixed with 20 ml of silver nitrate stock solution drop by drop in a conical flask. The conical flask was wrapped with aluminum foil. The volume was adjusted by adding deionized water to 250 ml so that the final concentration of Ag + ion becomes 2 × 10^–3^ M and plant extract concentration remains 10% (v/v) of the stock solution. The mixture was then stirred with a magnetic stirrer at a temperature of 50 °C for 5 min, and then the flask was transferred to a thermostat water bath and warmed at 60 °C for 2 h. The transformation of the color of the solution into reddish brown from yellowish green was the first indication of the reduction of Ag^+^ to Ag^0^ form. The resultant reddish-brown colored solution was allowed to cool overnight in an air-tight, light-protected container. In a REMI Research Centrifuge instrument, the final nano-colloidal solution was centrifuged twice to eliminate un-interacted biological materials at 10,000 rpm for 15 min. The final pellet was collected and transferred to a vacuum desiccator for drying. Synthesized nanoparticles were stored properly for characterization and bioassays.

### Different analytical techniques used for the characterization of synthesized silver nanoparticles

Synthesized nanoparticles from *D. montana* were characterized by various scientific analytical methodologies. To detect the absorption maxima, the optical density of the reaction mixture was first scanned at a fixed wavelength range between 200 and 700 nm at regular intervals using a Multiskan GO spectrophotometer (version 1.00.40) with 1-nm resolution. Then, a dried pellet of nano-materials obtained after centrifugation was coated on XRD grid. The spectra were scanned by X-ray diffractometer (Rigaku, Smartlab) with a Cu Kα (I = 1.54A) radiation source. The scanning range was 10°–80°. An aliquot from the final pellet was used for field emission scanning electron microscopy (FE-SEM) integrated with energy dispersive X-ray spectroscopy (EDS). For structural characteristics of synthesized nanoparticles, images from a transmission electron microscope (TEM) were obtained using the instrument of JEOL; JEM 1400 plus operated at 200 kV. A sample for TEM analysis was prepared by dropping the aqueous AgNPs on carbon-coated copper grids (300 mesh sizes) and then drying them in a vacuum at 25 °C overnight. Clear nano solution, prepared by repeated centrifugation of the reaction mixture, was used for Malvern's dynamic light scattering (DLS) and zeta potential analyzer. Dried powder of nano-materials was used for FTIR measurements to obtain an idea about the chemical framework around the synthesized nanoparticles by an FT-IR Spectrometer (Perkin Elmer Lx10-8873). The scanning range was 450–4000 cm^−1^ at a resolution of 4 cm^−1^.

### Mosquitocidal bioassays

The efficacy of synthesized nanoparticles was tested on immatures (larvae and pupae) of *Ae. albopictus* according to the standard protocol specified by WHO^33^. Twenty-five larvae of each instar (I-IV) and pupae were taken in separate glass beakers filled with 100 ml of double distilled water. Six concentrations of nano solution (0.3125, 0.6250, 1.2500, 2.5000, 5.0000, and 10.0000 ppm) were used to examine larvicidal and pupicidal efficacies. 0.5 mg larval food was provided in each glass beaker. Three trials for each concentration against each larval instar and pupae were performed in an experimental setup. The experimental setup was replicated on three different days (i.e., n = 9). A control using only distilled water was maintained for each concentration during experimentation. Mortality of immature was recorded after 24, 48, and 72 h of exposure. Control mortality was corrected by Abbott's formula^34^.

$$\mathrm{Percentage \, mortality}=\frac{\mathrm{Number \, of \, larvae\, }/\mathrm{ pupae \, introduced}-\mathrm{Number \, of \, larvae\, }/\mathrm{ pupae \, alive}}{\mathrm{Number \, of \, larvae\, }/\mathrm{ pupae \, introduced }}\times 100$$

### Examination of the length of larvicidal efficacy of synthesized nanoparticles up to three and half years

An experiment was done for up to three and a half years to determine the stability of synthesized silver nanoparticles at an interval of three months. Twenty-five 4th instar *Ae. albopictus* larvae were kept in a 250 ml glass container filled with 100 ml of distilled water. A five ppm concentration of synthesized AgNPs was used for that long-term bioassay. The experiment was triplicated in three separate containers for more accuracy. After that experiment, DLS analysis and Zeta potential measurement were done to test the stability of synthesized silver nanoparticles.

### Test on non-target organisms

**Figure 2:**
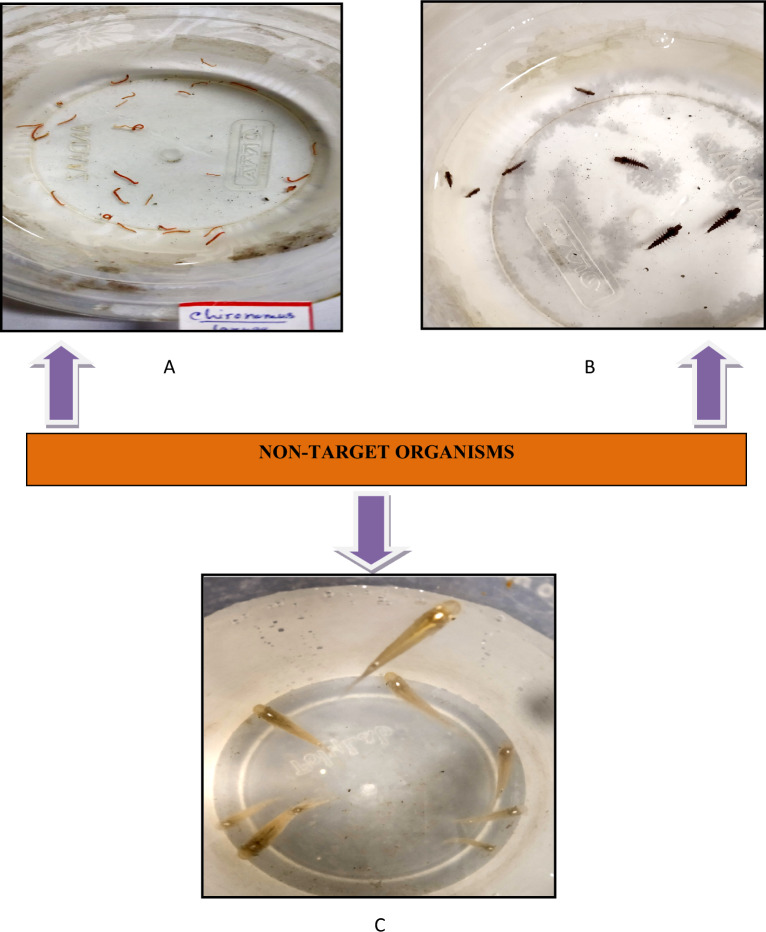
Pictures of three non-target organisms (**A**) *Chironomus circumdatus*, (**B**) *Toxorhynchites splendens,* and (**C**) *Aplocheilus panchux.*

Synthesized nanoparticles using *D. montana* were tested for any toxicity and abnormalities against some non-target organisms following the same protocol. Five concentrations (0.6250, 1.2500, 2.5000, 5.0000, and 10.0000 ppm) were used for the experiment against non-target species. *Toxorhynchites splendens* larvae (mosquito predator), *Chironomus circumdatus* larvae (chironomid), and *Aplocheilus panchax,* a mosquito larvivorous fish, were used as non-target organisms as they share common habitats with mosquito larvae (Fig. 2).

### Data analysis

Larval and pupal mortality were subjected to probit analysis^35^. The LC_90_ values, regression equations (Y = mortality; X = concentrations), and regression coefficient values were computed using the software "STAT PLUS 2007 (Trial version)" and MS EXCEL 2003. The Abbott׳s formula was used to adjust the control mortality percentage.

## Results

### Determination of plant compounds

#### Phytochemical analysis

Qualitative phytochemical analysis of the aqueous plant extract revealed the presence of alkaloids, flavonoids, terpenoids, steroids, saponins, coumarin, glycosides, etc. (Table 1). These secondary metabolites can reduce metal salts.

**Table 1:** Preliminary qualitative phytochemical screening of leaf extract of *Diospyros montana.*

Phytochemicals	Results
Alkaloid	**+**
Flavonoids	**+**
Tannins	**–**
Terpenoids	**+**
Steroids	**+**
Glycosides	**+**
Saponins	**+**
Coumarin	**+**

**‘+’ = **present, **‘–’ = **absent.

#### Determination of reducing and stabilizing performances of Plant Compounds

**Figure 3:**
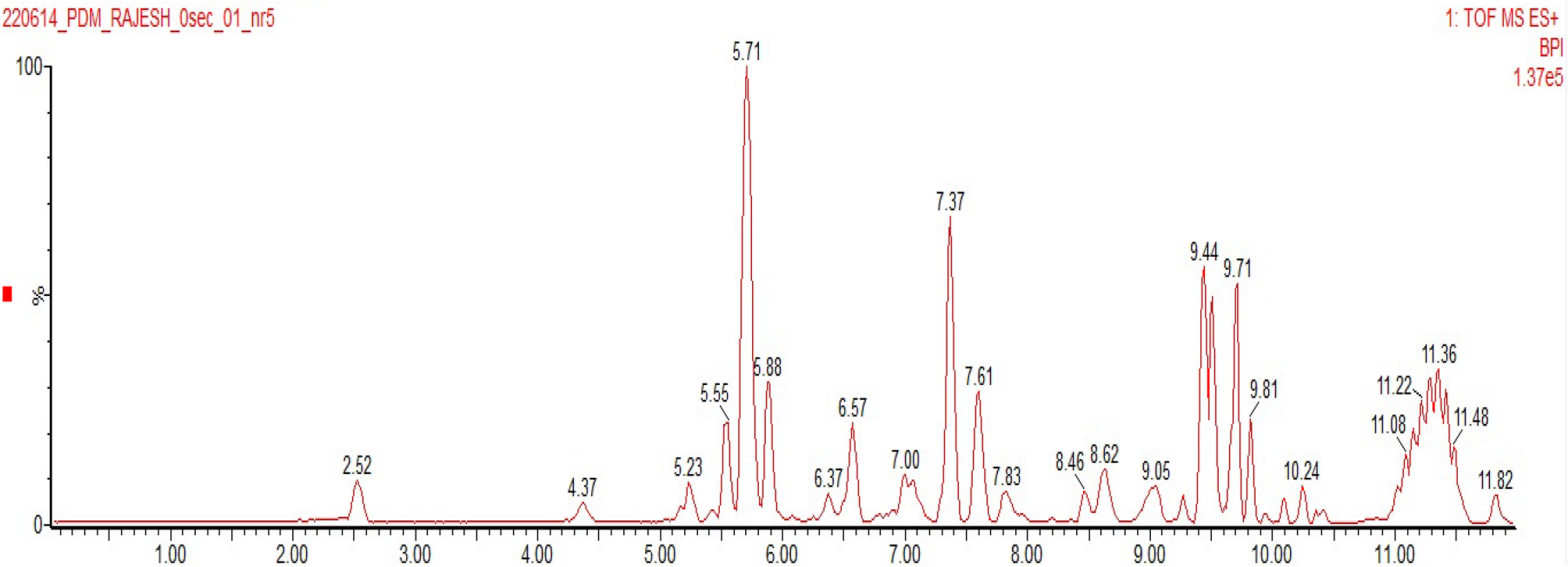
LC–MS chromatogram of leaf extract of *Diospyros montana.*

**Figure 4:**
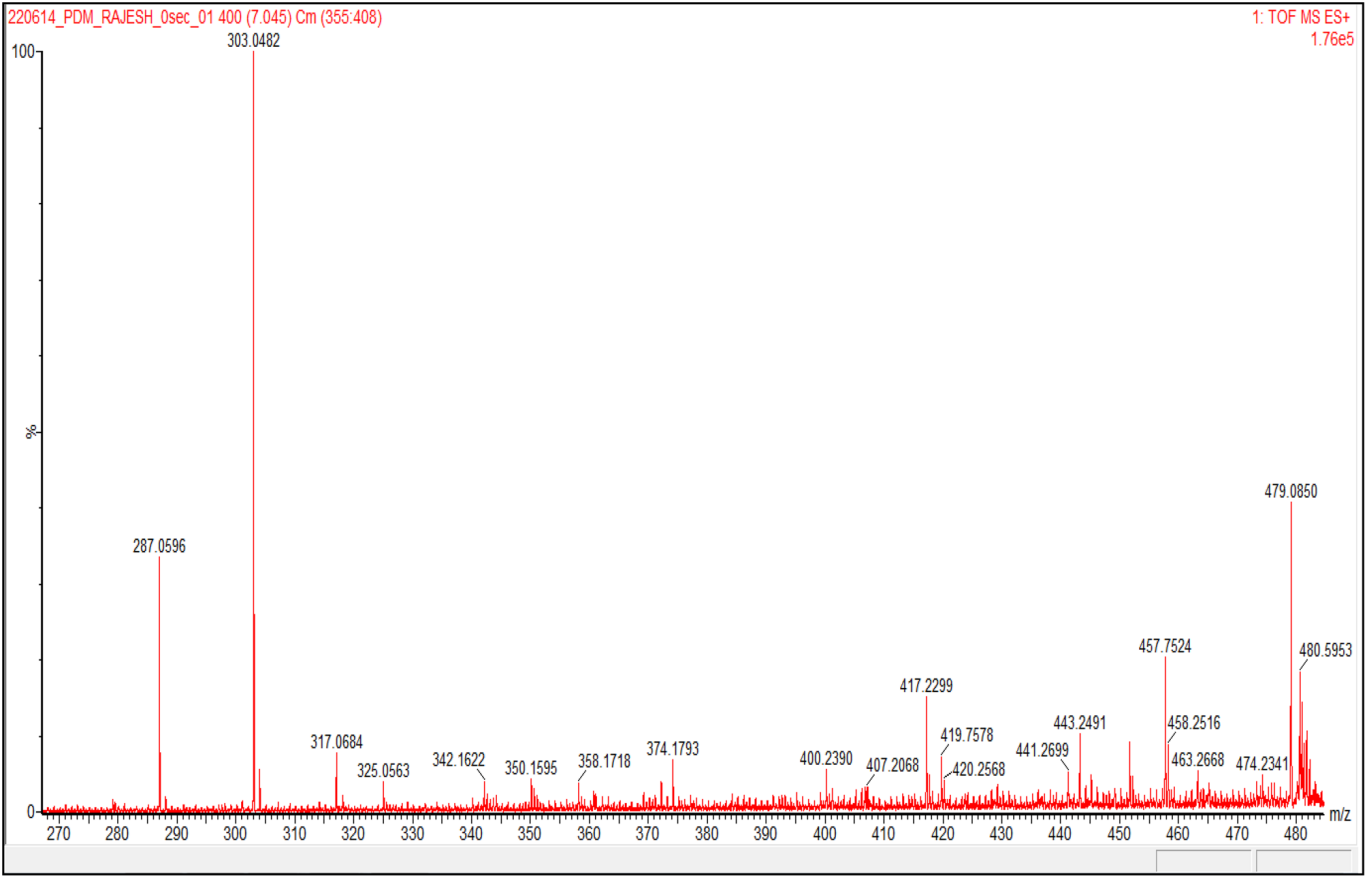
LC–MS spectrum of leaf extract of *Diospyros montana.*

To determine the phytochemical compounds of *D. montana* responsible for reduction and stabilization during the synthesis of silver nanoparticles, Liquid chromatography-mass spectrometry (LC–MS) analysis of the leaf extract was done. Figure 3 represents the LC–MS chromatogram of the leaf extract of *D. montana*. LCMS spectrum revealed flavonoid compounds, including quercetin (303.05 Da.), luteolin (287.05 Da.), kaempferol (287.11 Da.), and other polyphenol compounds, including Gallocatechin gallate (458.25 Da.) and Epigallocatechin gallate (458.25 Da.) and also alkaloid compound like capsaicin (306.15 Da.) were present in the leaf extract of *D. montana* (Fig. 4).

### Characterization of synthesized silver nanoparticles

#### UV–VIS spectroscopy study

**Figure 5:**
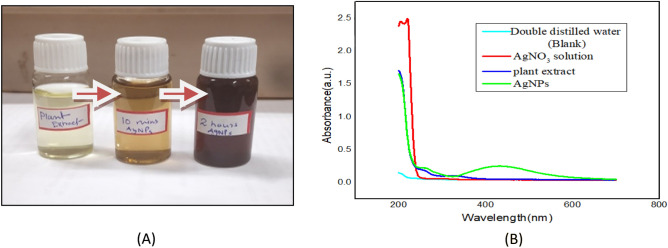
(**A**) The solution of diluted silver nanoparticles exhibits color change. (**B**) Distinctive surface plasmon absorption spectrum of silver nanoparticles (AgNPs) synthesized using leaf extract of *Diospyros montana*, with a maximum wavelength of 430 nm.

Changes in color from yellowish green to reddish brown occurred due to the excitation of surface plasmon vibrations in metal nanoparticles. Here, the characteristic surface plasmon absorption spectral band is shown at (max) 430 nm (Fig. 5B). The absorbance of the AgNPs solution showed (color change) after 2 h in Fig. 5A. 20 ml of aqueous 10^–3^ M AgNO_3_ solution was exposed to sunlight for the blank analysis and subjected to UV–vis spectroscopy study. It was observed that there was no color change in the aqueous AgNO_3_ solution since nanoparticles were not synthesized.

#### X-ray diffraction analysis

**Figure 6:**
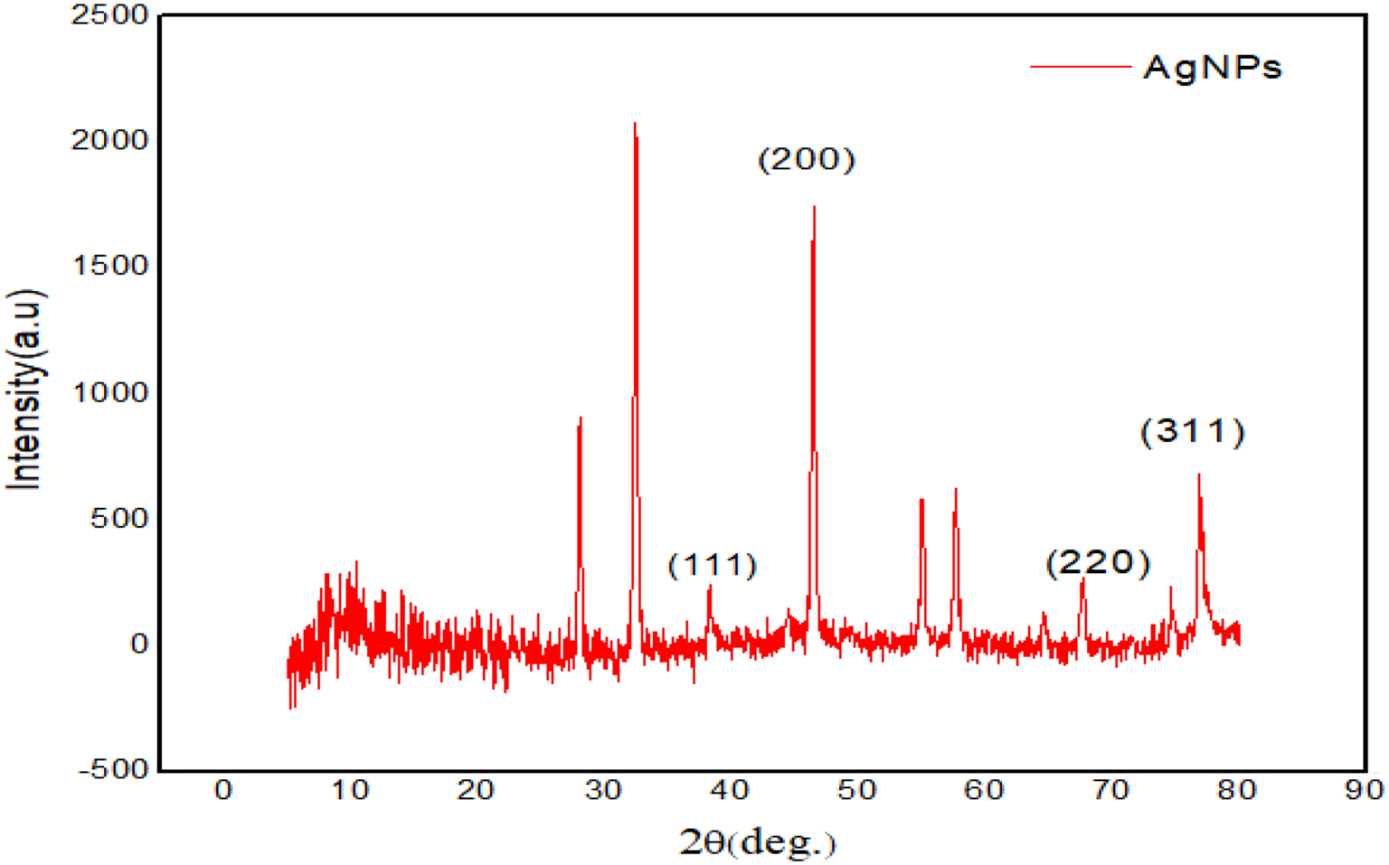
X-ray Diffraction pattern of the silver nanoparticles synthesized using leaf extract of *Diospyros montana*.

Any crystal that receives an X-ray reflection produces a variety of diffraction patterns, which are representations of the physicochemical properties of the crystal formations. Here, diffraction peaks were found at corresponding 2θ vales of 38.4°, 44.45°, 64.7°, and 77.15° those were attributed to the four different facets known for zero-valent face-centered cubic silver, which represented the (111), (200), (220), and (311) crystal planes respectively (Fig. 6). Additionally, a few unassigned peaks at 32.5°, 55°, and 57.67° were seen close to the typical peaks. These distinct Bragg peaks could result from the capping agent's stabilizing effects on the nanoparticles. Analysis was made by comparing the diffracted beams with the reference database in the Joint Committee on Powder Diffraction Standards (JCPDS) library.

#### FE-SEM and EDX Analysis

**Figure 7:**
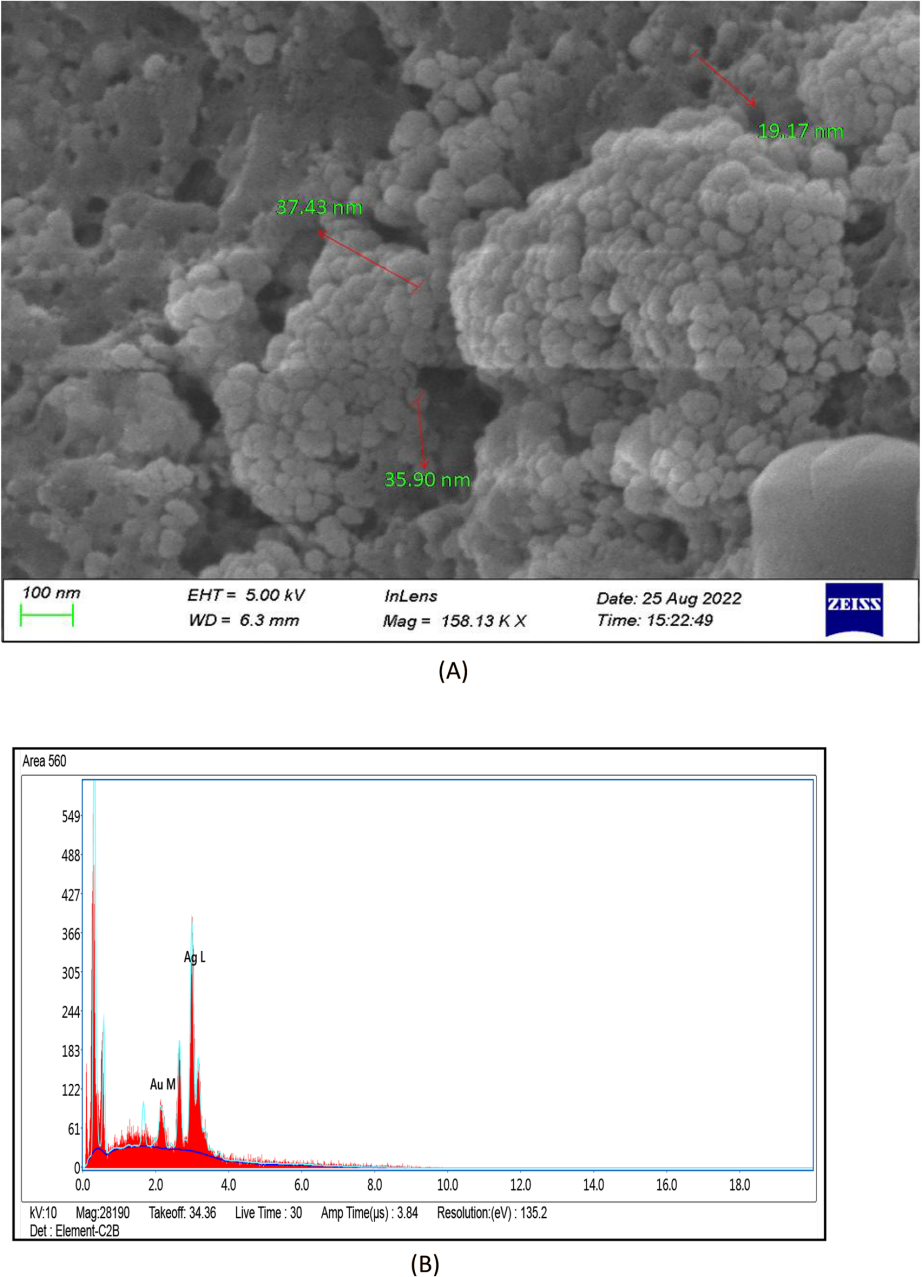
(**A**) Field emission scanning electron microscopy images of silver nanoparticles (AgNPs) synthesized by *Diospyros montana* (**B**) EDX spectrum of synthesized silver nanoparticles (AgNPs) using leaf extract of *Diospyros montana*.

The produced nanoparticles in the SEM micrograph had spherical and semi-spherical structural features (Fig. 7A). Energy-dispersive X-ray spectroscopy (EDX) combined with FE-SEM could be used to examine silver powder morphology and also perform chemical composition analysis. The outcome showed that pure AgNPs were present in the reaction product. EDX spectrum exhibited an optical absorption peak at about 3 keV. The significant signal in the silver region of the EDX spectrum provided evidence that silver particles were formed (Fig. 7B). 89.43% yield of silver element was determined through EDX analysis.

#### TEM analysis

**Figure 8:**
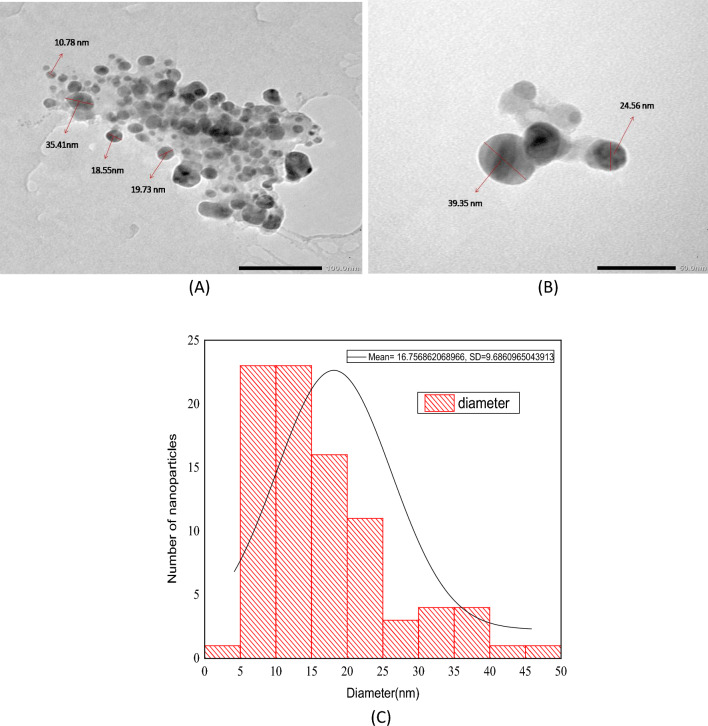
(**A**) Representative Transmission Electron Microscopy photograph of silver nanoparticles (AgNPs) synthesized using leaf extract of *Diospyros montana*. (**B**) Closer view of the polyhedral shape of silver nanocrystals. (**C**) Size distribution histogram of silver nanoparticles synthesized using leaf extract of *Diospyros montana*.

Transmission electron microscopy (TEM) is a valuable, widely used, and essential technology to get precise measurements of particle size, size distribution, and shape of nanoparticles. TEM image of synthesized nanoparticles presented in Fig. 8A 100 nm and B 50 nm scale imparted that the majority of newly generated nanocrystals had a quasi-spherical (or polyhedral) shape. Some were irregular in shape. The average size of synthesized AgNPs determined through the TEM image was 16.75 nm (Fig. 8C).

#### Dynamic light scattering (DLS) and zeta potential analysis

**Figure 9:**
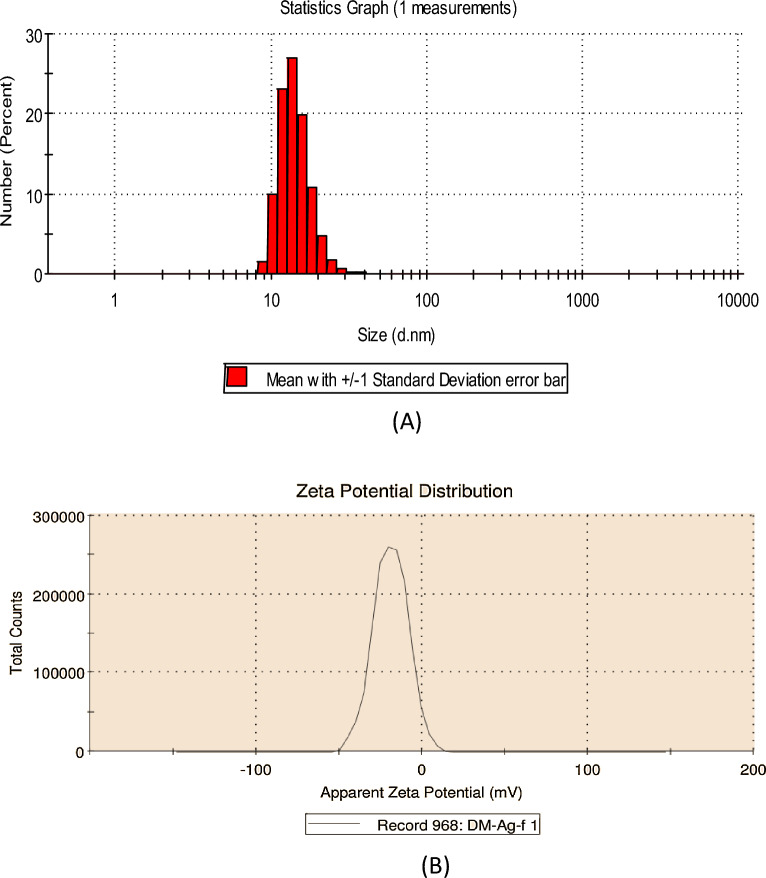
(**A**) Particle size histogram of silver nanoparticles (AgNPs) synthesized using leaf extract of *Diospyros montana.* (**B**) Zeta potential chromatogram of silver nanoparticles (AgNPs) synthesized using leaf extract of *Diospyros montana.*

DLS makes it possible to calculate the hydrodynamic size of particles by analyzing the variation of the scattered light intensity as a function of time. The size obtained from DLS was larger than TEM, which might be due to the influence of Brownian motion. Size distribution through DLS analysis of nanoparticles synthesized using *D. montana* is presented in Fig. 9A. Particle size ranges from 8.72 to 50.75 nm. Supplementary Table 1 illustrates the size distribution of synthesized AgNPs through percent values. The Zeta potential of the nano solution was also measured. The Zeta potential of the nano solution was highly negative with a value of minus nineteen, approximately (− 19.10) mV. The zeta potential value is presented in Fig. 9B.

#### FT-IR analysis

**Figure 10:**
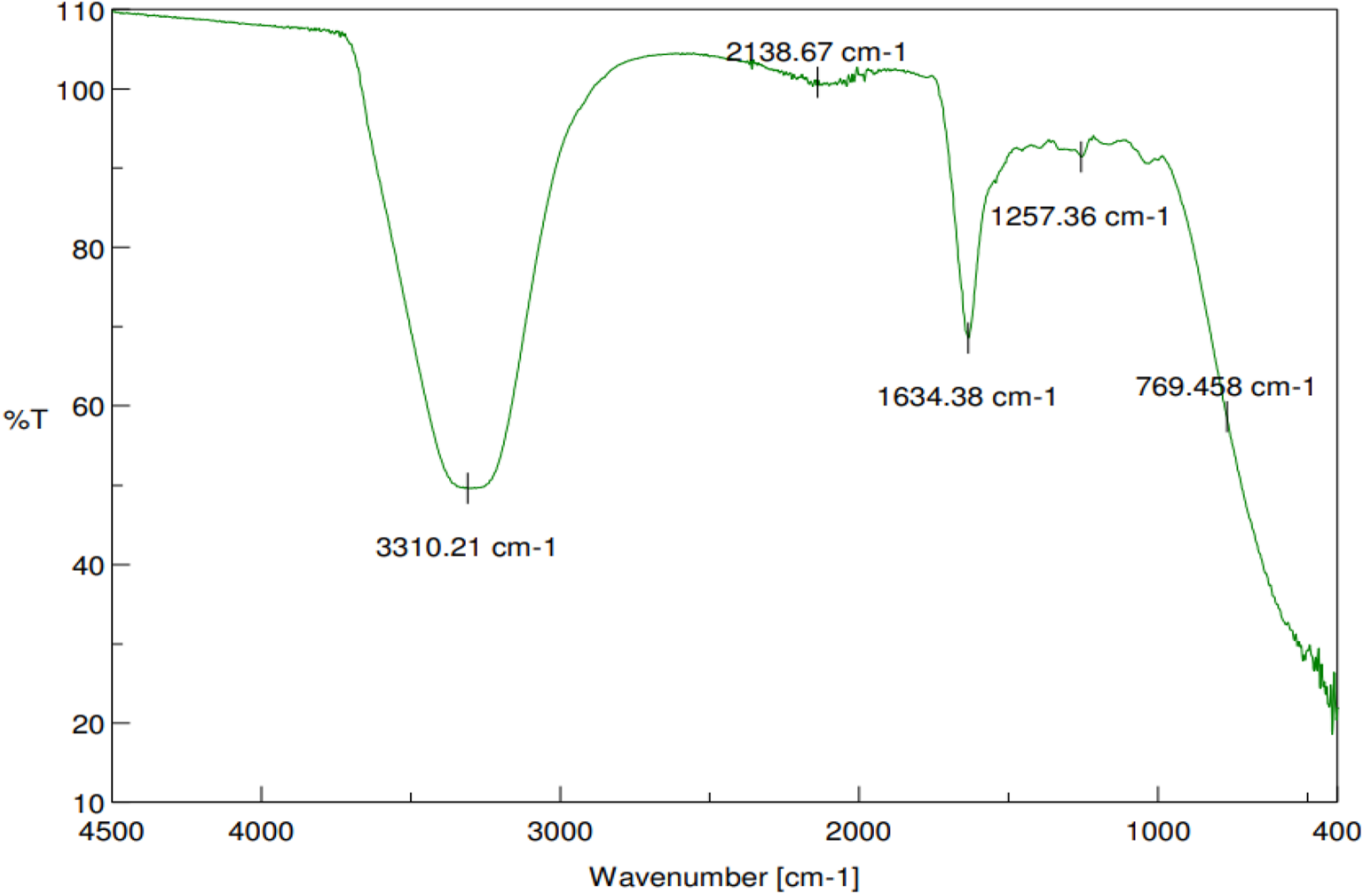
FTIR spectrum of silver nanoparticles synthesized by *Diospyros montana* leaf extract.

FTIR spectrum of biosynthesized silver nanoparticles (Fig. 10) revealed distinct peaks at 3310.21 cm^−1^, 2138.67 cm^−1^, 1634.38 cm^−1^, 1257.36 cm^−1^, and 769.45 cm^−1^. Each of these bands represents different functional groups, which are interpreted in Table 2.

**Table 2:** FTIR absorption spectra bands of synthesized silver nanoparticles and their probable functional groups.

Absorption spectra peaks	Range (cm^−1^)	Probable functional groups
3310.21 cm^−1^	3270–3320	C=O and N–H stretching of amide
	3200–3400	O–H stretching of alcohol
2138.67 cm^−1^	2100–2140	C≡C stretching of alkynes
1634.38 cm^−1^	1630–1680	C=O stretching of ketones and amide
	1620–1680	C–C stretching of alkenes
	1630–1680	C=O stretching of amide
1257.36 cm^−1^	1180–1260	C=O stretching of alcohol
	1200–1305	N–H stretching of amide
769.45 cm^−1^	1000–1100	N–H stretching of amine

### Mosquitocidal bioassay study

The percent mortality of mosquito immatures subjected to aqueous solutions containing pure AgNPs pellets at various concentrations (0.3125, 0.6250, 1.2500, 2.5000, 5.0000, and 10.0000 ppm) for 72 h is shown in Table 3. Cent percent mortality has been noted at a 10 ppm concentration of nano-material after 48 h of exposure against all larval instars. In pupicidal bioassay, 100% mortality was found at 10 ppm after 72 h of exposure. The mortality of mosquito immatures (larvae and pupae) increased with increasing concentration of nano solution and time exposure. The LC_90_ values, regression equations, and R^2^ values of synthesized Ag NPs against immures of *Ae. albopictus* are depicted in Table 4. Table 5 shows the outcomes of three-way factorial ANOVA of mortality with three fixed variables: larval instar, extract concentration, and exposure hour.

**Table 3:** Larvicidal and pupicidal activity of *Diospyros montana* induced green synthesized silver nanoparticles (AgNPs) on the larvae and pupae of *Aedes albopictus.*

Larval instars	Concentrations (ppm)	Mortality percentage (mean ± SE)		
		24 h	48 h	72 h
FIRST	0.3125	60.44 ± 5.51	72.44 ± 4.29	86.22 ± 2.50
	0.6250	74.22 ± 5.54	86.22 ± 4.11	94.22 ± 2.32
	1.2500	76.88 ± 5.48	87.55 ± 3.42	100.00 ± 0.00
	2.5000	89.77 ± 3.77	100.00 ± 0.00	100.00 ± 0.00
	5.0000	100.00 ± 0.00	100.00 ± 0.00	100.00 ± 0.00
	10.0000	100.00 ± 0.00	100.00 ± 0.00	100.00 ± 0.00
	Control	0.00 ± 0.00	0.00 ± 0.00	0.00 ± 0.00
SECOND	0.3125	52.00 ± 5.81	64.88 ± 4.79	69.33 ± 4.32
	0.6250	62.66 ± 5.65	77.77 ± 4.42	90.66 ± 2.66
	1.2500	67.55 ± 3.01	83.55 ± 3.42	94.22 ± 2.01
	2.5000	81.77 ± 6.39	89.33 ± 5.03	95.11 ± 2.81
	5.0000	100.00 ± 0.00	100.00 ± 0.00	100.00 ± 0.00
	10.0000	100.00 ± 0.00	100.00 ± 0.00	100.00 ± 0.00
	Control	0.00 ± 0.00	0.00 ± 0.00	0.00 ± 0.00
THIRD	0.3125	18.22 ± 2.22	34.22 ± 2.41	49.77 ± 2.83
	0.6250	45.77 ± 3.34	65.33 ± 3.05	79.11 ± 3.03
	1.2500	54.66 ± 4.66	73.77 ± 2.32	87.55 ± 2.35
	2.5000	62.66 ± 5.49	75.55 ± 4.29	88.00 ± 2.58
	5.0000	86.22 ± 2.50	92.44 ± 2.53	96.44 ± 1.04
	10.0000	95.11 ± 1.29	100.00 ± 0.00	100.00 ± 0.00
	Control	0.00 ± 0.00	0.00 ± 0.00	0.00 ± 0.00
FOURTH	0.3125	12.88 ± 1.85	19.55 ± 1.81	25.33 ± 1.15
	0.6250	34.66 ± 3.12	44.88 ± 3.31	53.77 ± 2.75
	1.2500	42.22 ± 2.75	56.44 ± 3.42	71.55 ± 2.86
	2.5000	58.22 ± 5.81	68.88 ± 4.65	82.66 ± 2.21
	5.0000	78.22 ± 5.85	89.33 ± 3.33	96.44 ± 1.55
	10.0000	95.55 ± 1.23	100.00 ± 0.00	100.00 ± 0.00
	Control	0.00 ± 0.00	0.00 ± 0.00	0.00 ± 0.00
PUPA	0.3125	7.11 ± 0.88	15.55 ± 1.23	22.22 ± 1.17
	0.6250	21.77 ± 2.50	30.66 ± 2.58	38.22 ± 2.41
	1.2500	45.77 ± 2.22	58.66 ± 2.40	71.11 ± 2.08
	2.5000	67.55 ± 2.15	84.88 ± 1.60	92.44 ± 1.55
	5.0000	85.77 ± 1.77	97.33 ± 0.94	99.11 ± 0.58
	10.0000	94.66 ± 1.15	98.66 ± 0.66	100.00 ± 0.00
	Control	0.00 ± 0.00	0.00 ± 0.00	0.00 ± 0.00

*S.E* Standard error, *ppm* parts per millions.

**Table 4:** Assessment of LC_90_ values of synthesized silver nanoparticles on *Aedes albopictus* larvae through log-probit and regression analyses.

Larval instars	Period of exposure (hour)	LC_90_	SE	LCL	UCL	Regression equation	R^2^ value
FIRST	24	1.987	0.621	1.156	6.386	y = 3.506x + 72.04	0.678
	48	0.900	0.242	0.526	2.315	y = 2.091x + 84.17	0.483
	72	0.3997	0.053	0.301	0.506	y = 0.818x + 94.05	0.288
SECOND	24	2.9910	1.446	1.537	20.507	y = 4.684x + 61.95	0.753
	48	1.6873	0.249	1.314	2.340	y = 2.984x + 76.12	0.663
	72	0.8721	0.116	0.686	1.157	y = 1.912x + 85.27	0.382
THIRD	24	7.5227	1.237	5.697	10.826	y = 6.572x + 38.87	0.760
	48	3.8676	1.390	2.135	15.093	y = 5.047x + 56.99	0.654
	72	1.9473	0.276	1.532	2.669	y = 3.393x + 72.34	0.484
FOURTH	24	9.2699	1.473	7.0716	13.154	y = 7.494x + 29.03	0.851
	48	5.1974	1.220	3.259	11.867	y = 6.982x + 40.26	0.769
	72	3.0254	0.357	2.467	3.919	y = 5.963x + 52.05	0.610
PUPA	24	6.6435	0.808	5.376	8.652	y = 8.132x + 27.08	0.738
	48	3.5041	0.363	2.923	4.387	y = 7.526x + 39.59	0.624
	72	2.3244	0.226	1.962	2.873	y = 6.667x + 48.63	0.551

*LC*_*90*_ Lethal concentration (to kill 90% of exposed population), *SE* standard error, *LCL* Lower control limit, *UCL* Upper control limit.

**Table 5:** Three-way ANOVA analysis of mortality of *Aedes albopictus* using green synthesized silver nanoparticles using *Diospyros montana* leaf extract.

Source of variation	Sum of square	df	Mean square	F value	p-level	F crit. value	Omega square
Instars (I)	103,409.97	4	25,852.49	332.87	0	4.66	0.17
Concentrations (C)	328,429.43	5	65,685.88	845.76	0	4.15	0.54
Hours (H)	39,569.81	2	19,784.90	254.74	0	4.15	0.06
I × C	59,793.58	20	2989.67	38.494	0	2.30	0.09
I × H	1676.00	8	209.50	2.69	0.006	3.31	0.00
C × H	9987.04	10	998.70	12.85	0	3.00	0.01
I × C × H	3400.61	40	85.01	1.09	0.32 (N.S)	1.09	0.00
Within groups	55,918.22	720	77.66				
Total	602,184.69	809	744.35				
Omega squared for combined effect	0.89						

Significant level *p* < 0.05.

*df* Degree of freedom, *N.S* Not significant.

### Examination of the duration of larvicidal efficacy of synthesized nanoparticles

**Figure 11:**
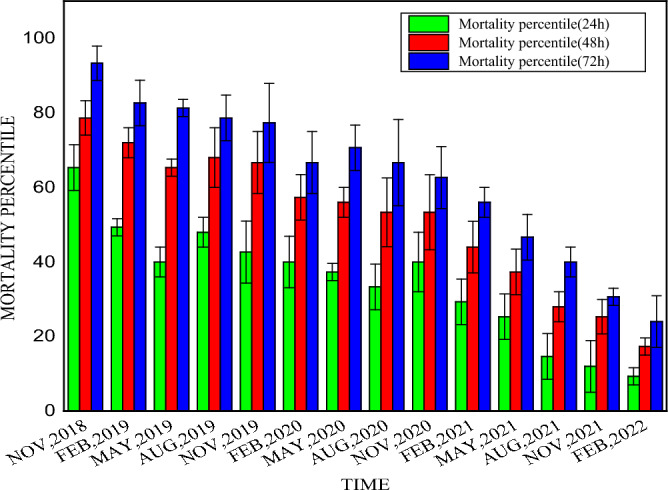
Mortality percentiles in long-term efficacy (stability) test of synthesized silver nanoparticles (AgNPs).

**Figure 12:**
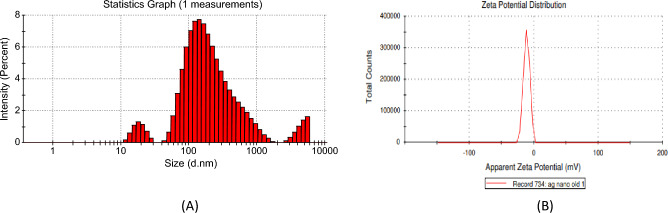
(**A**) DLS analysis and (**B**) Zeta potential measurement of synthesized silver nanoparticles after three and half year of synthesis.

The duration of larvicidal efficacy examined up to three and half years is presented in Fig. 11. Again, remarkable stability in larvicidal effectiveness was recorded with a prolonged decreasing trend. In November 2018, the mortality was 93.33%. In November 2019, mortality decreased by 16–77.33%. In November 2020, mortality decreased by 30.67–62.66%. In November 2021, mortality decreased by 62.66–30.66%. Lastly, up to February 2022, mortality was reduced by 69.33%. So, *Aedes albopictus* larvae mortality gradually decreased to 24% in three and a half years. DLS analysis of synthesized silver nanoparticles after that long duration showed that the particles were relatively larger because of their aggregation. The Zeta potential value of synthesized AgNPs increased to minus eleven millivolts, approximately (− 11.3 mV) (Fig. 12).

### Effect on non-target organisms

The effect of synthesized AgNPs on three non-target organisms, *Toxorhynchites splendens* larvae, *Chironomus circumdatus* larvae, and *Aplocheilus panchux* were presented in Table 6. LC_90_ values of synthesized silver nanoparticles against *Toxorhynchites splendens* larvae and *Chironomus circumdatus* larvae are presented in Table 7. When treated on *Aplocheilus panchux* fish, synthesized silver nanoparticles showed the least mortalities at the highest concentration. But in lower concentrations, *Aplocheilus panchux* didn't show any mortality.

**Table 6:** Mortality of non-target organisms on different concentrations of synthesized silver nanoparticles (AgNPs).

Non–target organisms	Concentrations (ppm)	Mortality percentage (mean ± SE)		
		24 h	48 h	72 h
*Toxorhynchites splendens* larvae	0.6250	0.00 ± 0.00	0.00 ± 0.00	0.00 ± 0.00
	1.2500	2.66 ± 1.33	6.66 ± 1.33	8.00 ± 0.00
	2.5000	9.33 ± 1.33	10.66 ± 1.33	14.66 ± 1.33
	5.0000	10.66 ± 1.33	16.00 ± 2.30	18.66 ± 1.33
	10.0000	18.66 ± 3.52	24.00 ± 2.30	26..66 ± 1.33
	Control	0.00 ± 0.00	0.00 ± 0.00	0.00 ± 0.00
*Chironomus circumdatus* larvae	0.6250	0.00 ± 0.00	0.00 ± 0.00	0.00 ± 0.00
	1.2500	4.00 ± 2.30	8.00 ± 2.30	10.66 ± 1.33
	2.5000	8.00 ± 0.00	12.00 ± 2.30	16.00 ± 2.30
	5.0000	16.00 ± 2.30	21.33 ± 1.33	22.66 ± 2.66
	10.0000	20.00 ± 2.30	25.33 ± 1.33	29.33 ± 1.33
	Control	0.00 ± 0.00	0.00 ± 0.00	0.00 ± 0.00
*Aplocheilus panchax*	0.6250	0.00 ± 0.00	0.00 ± 0.00	0.00 ± 0.00
	1.2500	0.00 ± 0.00	0.00 ± 0.00	0.00 ± 0.00
	2.5000	0.00 ± 0.00	0.00 ± 0.00	0.00 ± 0.00
	5.0000	0.00 ± 0.00	0.00 ± 0.00	0.00 ± 0.00
	10.0000	6.66 ± 1.33	10.66 ± 2.66	13.33 ± 1.33
	Control	0.00 ± 0.00	0.00 ± 0.00	0.00 ± 0.00

*S.E* Standard error, *ppm* Parts per millions.

**Table 7:** Assessment of LC_90_ values of synthesized silver nanoparticles on *Toxorhynchites splendens* and *Chironomus circumdatus* larvae through log-probit analyses.

Non–target organisms	Period of exposure (hour)	LC_90_ (ppm)	SE	LCL	UCL
*Toxorhynchites splendens* larvae	24	569.87	819.04	141.23	13,298.48
	48	418.61	454.59	123.80	4948.64
	72	184.42	276.91	48.74	5288.25
*Chironomus circumdatus* larvae	24	451.97	532.27	127.43	6462.79
	48	417.55	439.75	124.88	4572.88
	72	349.58	330.74	112.57	3063.33

*LC*_*90*_ Lethal concentration (to kill 90% of exposed population), *SE* Standard error, *LCL* Lower control limit, *UCL* Upper control limit, *ppm* parts per millions.

## Discussion

The current study demonstrated for the first time how to make biogenic AgNPs and how effective they are at killing mosquitoes by using *Diospyros montana* leaf extract as a reducing and capping agent.

Quercetin, luteolin, kaempferol, gallocatechin gallate, epigallocatechin gallate, and capsaicin are among the novel reducing and capping agents found in *D. montana* leaf through LCMS analysis. These substances have the capacity to end-cap the AgNPs, greatly improving their ability to kill mosquito larvae. Lee and Park^36^ reported the role of quercetin as a reducing agent for the synthesis of silver and gold nanoparticles. According to Osonga et al.^37^, luteolin tetraphosphate (LTP) was the reducing and capping agent for AgNPs synthesis using water as the solvent. In 2018 Deng et al.^38^ reported kaempferol as a reducing and stabilizing compound for silver nanoparticle synthesis. Hussain and Khan^39^ reported Gallocatechin gallate and Epigallocatechin gallate as reducing agents for AgNPs synthesis. Amruthraj et al.^40^ described that an alkaloid compound, capsaicin, was a bio-reductant to reduce silver nitrate to form silver nanoparticles. In light of this, the plant metabolites identified in the current investigation utilizing LC–MS may function as reducing and capping agents in the formation of silver nanoparticles.

The production of biogenic AgNPs by reducing Ag^+^ ions into Ag^0^ is easily observed by visual observation (color change) and characteristic peaks in UV–Vis spectroscopy. In AgNPs, the conduction band and valence band lie very close to each other, in which electrons move freely. These free electrons give rise to a surface plasmon resonance (SPR) absorption band, arising due to the collective oscillation of electrons of AgNPs in resonance with the light wave^41^. The XRD data also suggests crystallization of the bio-organic phase on the surface of the silver nanoparticles, or vice versa. Generally, the broadening of peaks in the XRD patterns of solids is assigned to particle size effects^42^. The presence of broader peaks in the observed data can be attributed to the impact of the experimental conditions on the process of nucleation and growth of crystal nuclei. Additionally, these broader peaks suggest the existence of smaller particle sizes^43^. The size distribution pattern acquired through DLS method shows a moderate particle size range from 8.72 to 50.75 nm, of which the majority are found between 10 and 21 nm. A high negative value (− 19.10 mV) of zeta potential indicates the long-term stability of synthesized AgNPs and better colloidal properties due to electrostatic repulsion and high dispersity. SEM and TEM images depicted that the plurality of newly generated nanocrystals had a quasi-spherical (or polyhedral) and spherical shape. Typical optical absorption peak at about 3 keV due to surface plasmon resonance in EDX analysis confirmed the formation of metallic silver nanocrystals^44^. FTIR analysis verifies functional molecules covalently grafted onto silver nanoparticles or interactions between an enzyme and a substrate during catalysis^45^. The prominent peak at 3310.21 cm^−1^ revealed the O–H stretching in the produced sample, which might be caused by the presence of hydroxyl-containing substances like carboxylic acid, phenols, or alcohol. The carbonyl (C=O) group stretching was thought to be responsible for the peak at 1634.38 cm^−1^^46^. These results might point to proteins that could reduce and stabilize the silver nanoparticles. The apparent bands at 1257.36 cm^−1^ might reveal the stretching of the (C–O–C) group. The silver nanoparticles might be stabilized by Ag–O, Ag–N, or Ag-C bonding, which might be visible in the observable bands.

Dengue fever is a significant public health threat that affects the entire world and is carried by *Aedes* mosquitoes. It is indigenous to tropical and subtropical countries, especially in urban and suburban areas^47^. Earlier studies by different authors suggested that plant extracts, essential oils, etc., produced from plants and bacteria, especially distinct strains of *B. thuringiensis*, are potential alternative resources for mosquito control^48^. *D. montana*-derived green silver nanoparticles exhibit a promising mosquitocidal property. In earlier studies, Adhikari et al.^49^ revealed larvicidal properties of *Swietenia mahagoni-*derived silver nanoparticles against *Anopheles stephensi*, *Culex quinquefasciatus,* and *Culex vishnui* group with LC_90_ values of 55.34, 202.05, and 113.07 ppm, respectively after 72 h exposure. Suganya et al.^50^ showed the LC_90_ value of synthesized nanoparticles by using *Leucas aspera* against *Aedes aegypti,* which was 21.5685 mg L^−1^ after 24 h post-exposure. *D. montana-*derived nanoparticles are significantly more efficient regarding LC_90_ value for all instars larvae and pupae of the dengue vector, Asian tiger mosquito (*Ae. albopictus)*. The established regression equations clearly show a dose-dependent mortality, which is demonstrated by the positive correlation between the rate of mortality (Y) and the concentration of the AgNPs (X) and by the fact that each time the regression coefficient (R^2^) value is greater than zero. Outcomes of three-way ANOVA analysis revealed that different concentrations, instars, and times of exposure singly or in complex interaction significantly influenced the mortality of larvae and pupae because, in most cases, F values were greater than critical F values and p values were less than 0.05. Only the influence of one complex interaction (I × C × H) was not significant. The exact mechanisms of larvicidal and pupicidal effects of AgNPs are unknown. When applied to non-target organisms, synthesized silver nanoparticles showed LC_90_ values ranging from 184.42 to 569.87 ppm, but in the case of *Ae. albopictus* LC_90_ values were less than 10 ppm, indicating their target specificity. It is conceivable that the AgNPs penetrate the larval membrane and cause the sensitive larvae to perish. Previously, it was claimed that the passage of AgNPs through the membrane of a treated larva induced an interaction with cell molecules that led to the death of larvae^51^. Additionally, the enzymes were deactivated and produced peroxide once the AgNPs reached the midgut epithelial membrane, which caused cell death^52^. It is worth mentioning that nanoparticles synthesized during the present study are extremely stable and have long-lasting mosquitocidal effects that continue for years with a very slow diminishing efficacy.

## Conclusion

The results of the current study demonstrate that aqueous leaf extract of *D. montana* is a sustainable and environmentally acceptable source that can be employed as an efficient reducing and stabilizing agent for the production of exceptionally stable silver nanoparticles (AgNPs). The procedure used in this work to fabricate green synthesized AgNPs is safe, non-toxic, and environmentally acceptable. The biosynthesized AgNPs exhibit significant mosquitocidal properties against the Dengue vector *Ae. albopictus* at low concentrations. Biogenic AgNPs are not as hazardous to organisms that are not their intended targets, indicating the target-specificity of synthesized AgNPs. There may be an essential usage in various domains, particularly in vector control programs.

### Supplementary Information

Supplementary Tables.

## Data Availability

All data generated or analyzed during this study are included in this published article (and its supplementary information files).
