# Supplementary material for: Diospyros montana mediated reduction, stabilization, and characterization of silver nanoparticles and evaluation of their mosquitocidal potentiality against dengue vector Aedes albopictus
Source: Sci Rep. 2023 Oct 11;13:17202. doi: 10.1038/s41598-023-44442-7 (PMC10567741; doi:10.1038/s41598-023-44442-7)
Supplement: Supplementary file 1 — Supplementary Tables. [file 41598_2023_44442_MOESM1_ESM.pdf]

Diospyros montana mediated reduction, stabilization, and characterization of silver nanoparticles and evaluation of their mosquitocidal potentiality against dengue vector _Aedes albopictus_

Rajesh Kumar Malla[1], Goutam Chandra[1*]

[1]Mosquito Microbiology and Nanotechnology Research Units, Parasitology Laboratory, Department of Zoology, The University of Burdwan, Burdwan-713104, West Bengal, India.

E-mail: rajeshmallazoo@gmail.com

***Corresponding author:** Goutam Chandra **E-mail:** goutamchandra63@yahoo.co.in

SUPPLEMENTARY MATERIAL:

Supplementary Table 1**:** Particle size distribution obtained through DLS method

**Size diameter(nm)**	**Mean Number Percent**	**Size diameter(nm)**	**Mean Number Percent**	**Size diameter(nm)**	**Mean Number Percent**	**Size diameter(nm)**	**Mean Number Percent**
0.4	0	5.615	0	78.82	0	1106	0
0.4632	0	6.503	0	91.28	0	1281	0
0.5365	0	7.531	0	105.7	0	1484	0
0.6213	0	8.721	1.4	122.4	0	1718	0
0.7195	0	10.1	10	141.8	0	1990	0
0.8332	0	11.7	23.1	164.2	0	2305	0
0.9649	0	13.54	27	190.1	0	2669	0
1.117	0	15.69	19.9	220.2	0	3091	0
1.294	0	18.17	10.9	255	0	3580	0
1.499	0	21.04	4.8	295.3	0	4145	0
1.736	0	24.36	1.7	342	0	4801	0
2.01	0	28.21	0.6	396.1	0	5560	0
2.328	0	32.67	0.2	458.7	0	6439	0
2.696	0	37.84	0.1	531.2	0	7456	0
3.122	0	43.82	0.1	615.1	0	8635	0
3.615	0	50.75	0.1	712.4	0	1.00E+04	0
4.187	0	58.77	0	825	0		
4.849	0	68.06	0	955.4	0		
